# Mechanical force-induced manipulation of electronic conductance in a spin-crossover complex: a simple approach to molecular electronics†

**DOI:** 10.1039/d0na00285b

**Published:** 2020-05-14

**Authors:** Amrit Sarmah, Pavel Hobza

**Affiliations:** Institute of Organic Chemistry and Biochemistry of the Czech Academy of Sciences Flemingovo nam. 2 CZ-16610 Prague 6 Czech Republic amrit.sarmah@marge.uochb.cas.cz amrit.sarmah@uochb.cas.cz +420731015016; Department of Physical Chemistry, Palacký University CZ–77146 Olomouc Czech Republic

## Abstract

The atomic-scale technological sophistication from the last half-decade provides new avenues for the atom-by-atom fabrication of nanostructures with extraordinary precision. This urges the appraisal of the fabrication scheme layout for a modular nanoelectronic device based on an individual molecular complex. The mechanical force-induced distortion to the metal coordination sphere triggers a low-spin (LS) to high-spin (HS) electronic transition in the complex. The controlled structural distortions (relative to a specific bond-angle) are deemed to be the switching parameter for the observed spin-transitions. Mechanical stretching is the key to engineering a spin-state switch in the proposed molecular device. The spin-dependent reversible variation in the electronic conductance concurrent to the unique spin-states can be understood from the state-of-the-art Nonequilibrium Green's Function (NEGF) calculations. Combined with NEGF calculations, the DFT study further provides a qualitative perception of the electronic conductance in the two-terminal device architecture. From the transport calculations, there is also evidence of considerable fluctuation in the spin-dependent electronic conductance at the molecular junction with relative variations in the scattering limit. Subsequently, the present study shows significant advances in the transmission probabilities for the high-spin state of the Fe(ii) complex. The results empower the progress of nanoelectronics at the single molecule level.

## Abstract 2

The atomic-scale technological sophistication from the last half-decade provides new avenues for the atom-by-atom fabrication of nanostructures with extraordinary precision.

## Graphical abstract

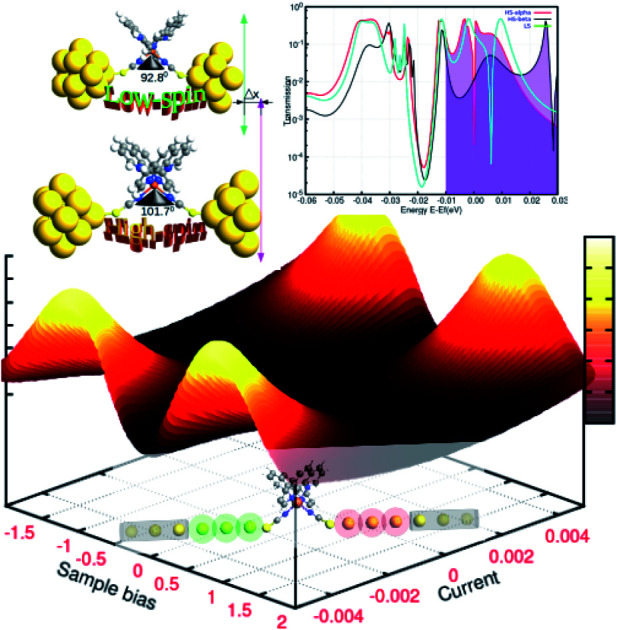

## Introduction

The rapid progress in measuring the *I*–*V* characteristics of single molecules or a cluster of small molecules opens new pathways in the prevailing momentum of molecular electronics.^1–5^ Briefly, the underlying concept of molecular electronics is to manipulate the function of an electronic component by tuning the electronic environment of a single molecule through external perturbations.^6^ In the reported literature, different examples of single-molecule switches are available.^7–10^ The precise control over the molecular conductance employing extraneous stimuli revolutionized electronics at the molecular level. The single-molecule system configured to molecular switches serves as a high-density memory device.^11^ Notably, the transition metal complexes with magnetic bistability are promising contenders as primitive building blocks of molecular electronics.

Certain metal complexes have intrinsic magnetic bistability, frequently attributed to the spin-crossover (SCO) complexes.^12^ The dynamic magnetic transition between a low-spin (LS) and high-spin (HS) state can be induced through temperature, light, pressure, magnetic or electric fields, or charge flow. The molecular conformation or structural orientation of the system is critical to retaining the energy equilibrium between the HS and LS states in SCO molecules. The switchable spin of the metal ion is crucial for the electronic transport in different spin-channels, producing instantaneous modulations to the overall conductance. Consequently, the realization of spin-dependent electronic transport at the molecular level has stimulated the unprecedented growth of molecular spintronics in recent years.^13^

The SCO system exhibits two distinct magnetic ground state configurations. Each spin-state remains stable up to certain external perturbations. Among the first-row transition metals, iron(ii) complexes are versatile and have been broadly investigated in this context.^14^ The existence of an octahedral ligand field environment splits the five iron d-orbitals into two levels. The electronic occupancies in these orbitals are controlled by two energy factors, the ligand field energy (*E*_LF_) and spin-exchange energy (*E*_EXC_). If *E*_LF_ ≫ *E*_EXC_, the electrons are all paired up at the T_2g_ level (total spin *S* = 0) and considered to be the low-spin (LS) state. The presence of external stimuli further disturbs the orbital degeneracy, resulting in an extensive separation of the electronic energy levels. Similarly, if *E*_EXC_ > *E*_LF_, the levels are occupied according to Hund's rule and results in a high-spin (HS) state (*S* = 2). Currently, it is possible to introduce controlled modulations to the existing *E*_EXC_/*E*_LF_ energy equilibrium in terms of different external stimuli. The dynamic magnetic transition between low and high-spin (HS) states can easily be achieved by bringing certain physical changes (*e.g.*, temperature, light, pressure), chemical changes (*e.g.*, covalent bonding), or by applying magnetic and electric fields. The non-covalent interaction-induced spin crossover was recently realized in the iron(ii) complex.^15^ It is also important to note that the LS-to-HS transition has a substantial impact on the molecular geometry, electronic structure, and HOMO–LUMO energy gap of the system in two different magnetic states.

**Fig. 1:**
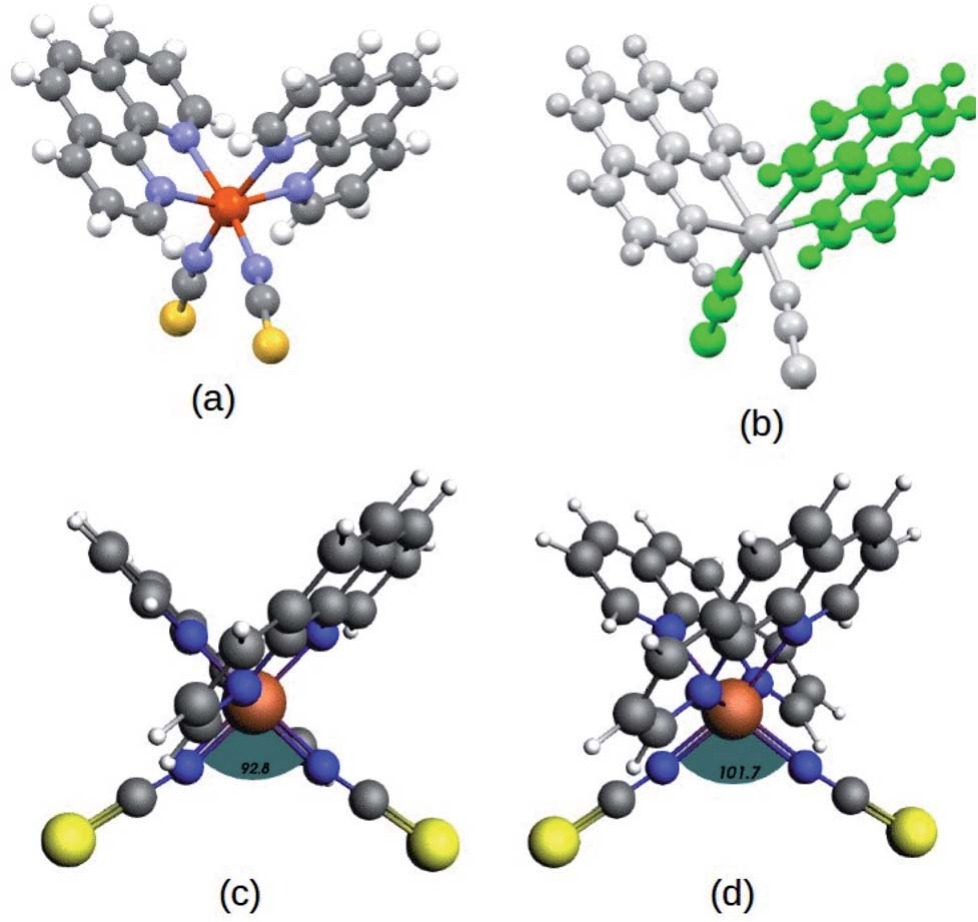
(a) Structure of the Fe(1,10-phenanthroline)_2_(NCS)_2_ molecule, (b) 2-fold symmetric orientation represented in different color shades, optimized structure of the complex in low-spin (LS) (c) and high-spin (HS) (d) states at the PBE(D3)/TZP level. The relative structural deformations in the two states are shown from the expansion in the ∠N–Fe–N bond angle from 92.80° (LS) to 101.70° (HS), as depicted in the figure.

The complex, Fe(1,10-phenanthroline)_2_(NCS)_2_ molecule, (Fe-phen) (shown in Fig. 1) has been well appreciated for its SCO behavior for a long time.^12^ The temperature-dependent spin-crossover (SCO) behavior of the complex was first predicted by Reiher *et al.*^16^ According to the report, the SCO phenomena are followed by considerable changes in the Fe–N bond distances and angles. As a result, there is a significant reduction in the ligand field strength around the central metal atom. Taking an analogy from the previous studies, we proposed a new methodology to manipulate the SCO in the Fe-phen complex with mechanical forces. The present study reports a simple device fabrication strategy based on the controlled manipulation of electronic conductance through a Fe-phen molecular junction. The conformational changes due to the mechanical stretching trigger the LS → HS transition in the Fe-phen molecule. We have performed DFT-based simulations to understand the correlation between the structural changes and the fluctuation in electronic conductance across the junction.

## Results and discussion

**Fig. 2:**
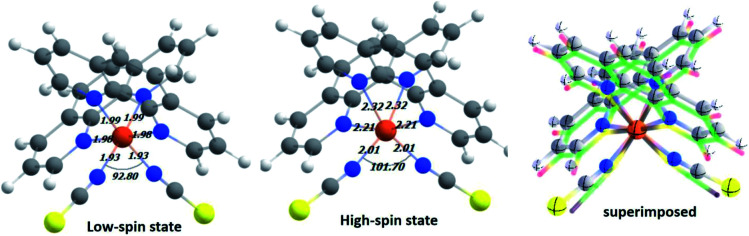
The optimized structure of the model system in low-spin (LS) and high-spin (HS) states at the PBE(D3)/TZP level. The important structural parameters around the Fe(ii) coordination sphere are reported here. A pictorial representation of the two superimposed structures is included for a better understanding of the relative structural changes between the different spin states.

In the first step, DFT-based calculations were performed to understand the electronic structure of the isolated Fe-phen complex. The optimized structures of the complex are shown in Fig. 2. We optimized the geometry of the complex at different spin states, starting from *S* = 0 (singlet) to *S* = 2 (quintet). The relative stability of the quintet (HS) state was found to be greater than the singlet state by ∼5.0 kcal mol^−1^, as evident from the optimized energy values. The graphical representations of the optimized energy components are available in the ESI.† Subsequently, we noticed considerable structural deformation associated with the low- to high-spin transition in the complex. Notably, there are several effective measures to ascertain the structural transformations in the Fe-phen complex in the LS and HS states. Here, we focused on the variation of the ∠N–Fe–N bond angle in the LS and HS states to satisfy the prerequisites of our device fabrication scheme. For the low and high-spin states, the particular bond angles were found to be 92.80° and 101.70°, respectively. For the convenience of our study, we will consider the shift in the ∠N–Fe–N bond angle value as the limiting criterion for the noted SCO. The application of mechanical force to compress or expand the two terminal sulfur atoms in the complex will alter the ∠N–Fe–N bond-angle, triggering an efficient spin-crossover in the system.

We witnessed a considerable increase in the metal–ligand bond lengths associated with the spin-flip from the singlet to quintet states. These inferences could be rationalized in terms of the one-particle picture with increasing antibonding molecular orbital occupation, and this correlated well to the previous report.^16^ Encouragingly, good agreement was found in the metal–nitrogen (phenanthroline) distances between theoretical and experimental results in both the singlet and quintet states. For the bond lengths, the small deviation from the experimental values (average ∼ 2 Å) represents reasonable accuracy for the computational method. A significant amount of distortion in the octahedral nitrogen atom environment of Fe(ii) in the HS-Fe(phen)_2_(NCS)_2_, correlated well with the experimental findings.^17^ Indeed, this is being pinpointed as the fundamental structural feature indicating the prominent role of the short-range (*i.e.*, local and non-lattice) structural changes.

It is worth mentioning here that the simple ligand field approximation adequately validated the observations from the DFT-based calculation. With a significant increase in the ligand field strength, the two bulky 1,10-phenanthroline ligands in the low-spin state exerts strong steric repulsion within a smaller periphery of the central Fe coordination sphere. The stronger metal–ligand interaction soon escalates the ligand field stabilization energy (LFSE), taking over the electronic pairing energy. This electron pairing at the highest occupied level favors the energetically stable low-spin state for the complex. On the other hand, mechanical stretching reduces the steric repulsion between the 1,10-phenanthroline ligands and disturbs the orbital degeneracy of the central metal atom. An effective reduction in the metal–ligand interaction decreases the ligand field strength. In this instance, the pairing of electrons becomes less favorable due to the substantial increase in pairing energy. This accounts for the feasibility of a high-spin state in the complex when the distance between the two sulfur atoms is significant.

**Fig. 3:**
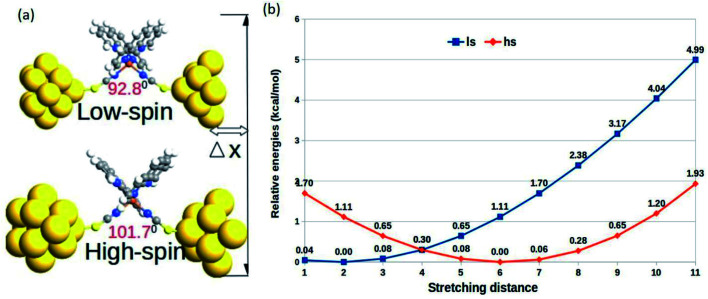
(a) The schematic representation of the mechanical stretching-induced SCO in the complex. (b) Variation in the relative optimized energies of the LS and HS states as a function of the stretching distance.

The relative changes in energy for the HS and LS states with respect to the gradual decrease in the sulfur–sulfur distance are shown in Fig. 3. According to our theoretical modeling, the distance between the two sulfur atoms in the low-spin (high-spin) optimized state at 7.65 Å (8.43 Å) was considered the starting point on the *x*-axis. It was observed that a relatively smaller separation between the sulfur atoms (*i.e.*, for a smaller value of ∠N–Fe–N angle) led to the energetically favorable LS state, while the regular increment in the sulfur–sulfur distance accounted for an energetically favorable HS state. A crossover point occurred with the metastable equilibrium between the LS and HS states when the distance between the two sulfur atoms was 4.0 Å. At this particular point, both spin states could coexist. A schematic representation of the spin-transition associated with the mechanical stretching of the model system is reported in Fig. 3. We included the electronic occupation and corresponding orbital energy order in the high and low-spin states of the complex in the ESI Sections S4 and S5,† respectively.

**Fig. 4:**
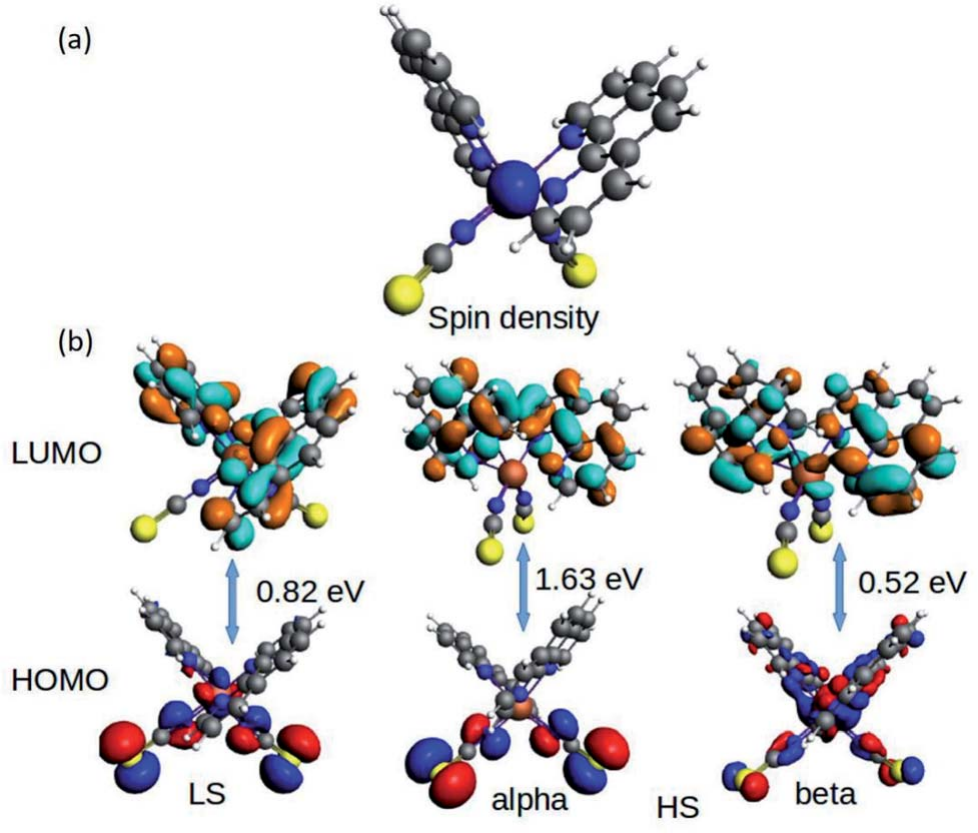
The isosurface of the (a) spin density and (b) HOMO–LUMOs for the Fe-phen complex in the LS and HS states.

**Fig. 5:**
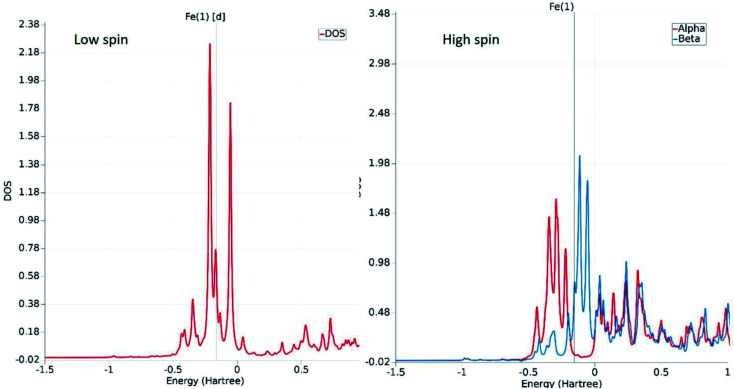
The relative positioning of PDOS for the d-orbitals of the Fe atom in the low-spin and high-spin states. The spin polarity of the electrons in the HS state is evident from the distinct appearance of the red (alpha) and blue (beta) plots near the Fermi level.

The fundamental chemical intuitions argued that the structure and orientation of the FMOs are critical to promote a better understanding of the electronic properties of a system. A comprehensive HOMO–LUMO study is essential to probe the adequate measures that trigger the spin-crossover in the model system. The characteristic FMO portrayals for the isolated complex (in gas-phase) for the two spin states are shown in Fig. 4. This indicates substantial changes in the position of the HOMO–LUMO lobes in the low and high spin states of the complex. The variations are more significant for the HOMOs compared to that of the LUMO. It is noted that the NCS ligands in the complex are the major contributors to the HOMO in the LS state of the complex. We observed a gradual increase in the contributions from the central metal atom, as well as the 1,10-phenanthroline ligands in the HOMO level at the HS state. Fig. 5 shows the partial density of states (PDOS) analysis of the central Fe atom that accounts for the d-orbital contribution to the overall density of states (DOS) of the system. As we have seen from the spin-density plot, the high-spin state is strongly spin-polarized. Here, an explicit PDOS analysis reveals that the d-electrons of the Fe center are primarily responsible for the induced spin-polarization in the high-spin state (Fig. 5).

The manipulation of individual atoms or particles with an explicit order during the process of fabrication is the underlying challenge and the immediate prerequisite for the evolution of nanoscale applications. In the single-molecule junction, electronic transport is regulated by the peripheral electronic architecture at the nanoscale region. This includes the molecule and a collection of metal atoms in the proximity of the molecule–metal contacts. The structural features around the molecule–metal contacts significantly perturbed the local electronic environment of the molecule. The electronic transport behavior is also profoundly sensitive to the unique number of feasible molecule–metal configurations and the nature of the metal atoms.

**Fig. 6:**
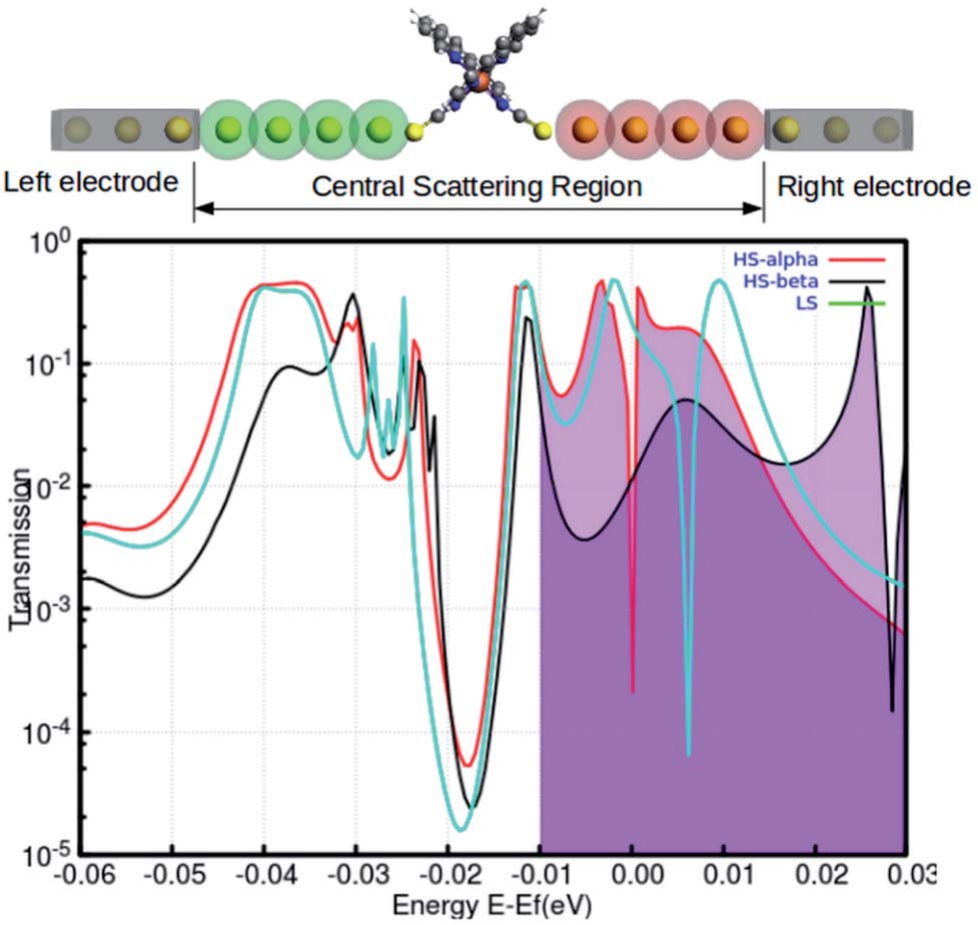
The total transmission of the LS and HS geometries shown in the atomic Au electrode based metal–molecule–metal configuration calculated with self-consistent DFT-NEGF. The two different color plots correspond to the spin-polarized transmission for up (red) or down (black) spin electron lines in the graph. The Fermi energy of the contacts is at 0 eV.

Recent reports have explored some unorthodox fabrication procedures to compose long and stable atomic chains.^18,19^ The concept employs a supporting substrate, such as a semiconductor or metal surface, as the template for compiling the atomic chains. In the modeled hybrid junction assembly, the metal–molecule–metal bridge is constructed by installing the Fe-phen molecule between 2 Au_3_ (consisting of three gold atoms in each chain) components (central scattering region) for the electronic transport. The semi-infinite atomic gold atoms are hooked up to the sulfur atoms of both NCS ligands in the complex. For this study, we adopted a TZP and SZ basis of numerical atomic orbitals to interpret the atoms on the Fe-phen molecule and the gold atoms, respectively. The NEGF-based transport calculations were performed at the GGA PBE(D3) level, as implemented in the ADF/band quantum chemistry package. It was presumed that the function of the mechanical forces (stretching) at the two edges of the gold electrodes would introduce structural deformation to the Fe-coordination sphere and lead to spin-crossover in the complex. Subsequently, there would be some effective modulations to the electronic conductance of the system in the HS and LS states. The visual graphics of the proposed device are represented in Fig. 6. The graphical illustration of the observed change in the electronic transmission of the system for the HS and LS states are shown in Fig. 6. The spin-polarized electronic transmission in the high-spin state is evident from the transmission *vs.* energy plot (red and black lines). Similarly, the plots demonstrate remarkable changes in the electronic transmission at the two spin states. It is worth mentioning here that the electronic behavior of a system around the Fermi level determines the versatility of that molecule for nanoscale device fabrication. As we observed from the plot (Fig. 5), the relative differences in the electronic transmission for the molecule in the two spin states are more pronounced around the Fermi level (the purple shaded zone). Furthermore, we observed a relative increase in the electronic transmission for the HS state in comparison with the LS state around the Fermi level. The metallic nature of one of the spin channels was estimated from the moderately high transmission (black line) across the Fermi level. However, the electronic transport through the other spin channel showed a significant decrease at the Fermi level. This seems to be an important implication for the electronic transport performance in the system. The theoretical findings support a higher probability of integrating the spin-polarized electronic transport through the Fe-phen molecular junction by unfolding the electrodes. It is important to understand that an experimental setup containing the AFM (atomic force microscopy) tool can effectively manipulate the structural distortion in the Fe-phen complex. Indeed, the SCO in the complex led to the evolution of spin-polarized electronic transport across the molecular junction, and outsourced the experimental realization of the proposed device fabrication.

**Fig. 7:**
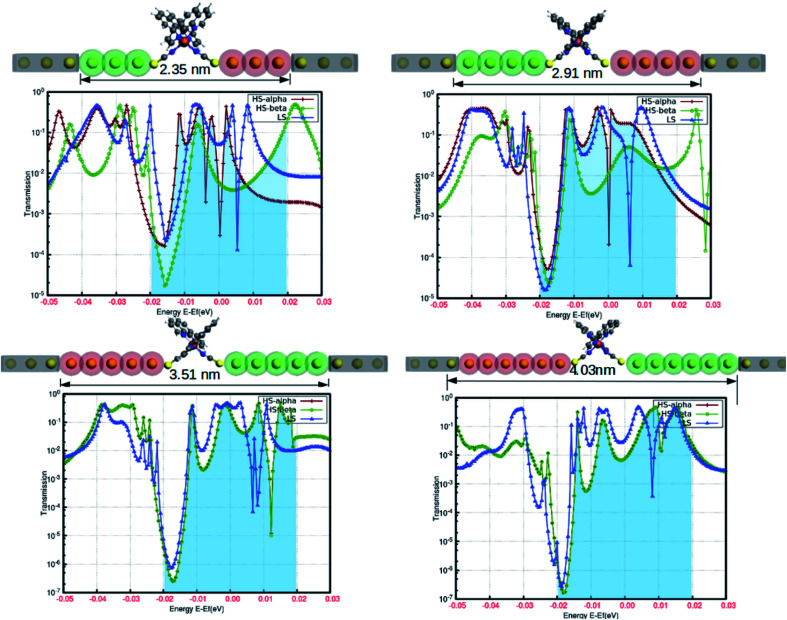
The relative variations in the electronic transmission by the gradual increase in the scattering region with the successive increment in the lead length (by atomic gold atoms). The important changes around the Fermi level are represented by the shaded regions in the plots.

In the simple assumption, electron transport is regarded as the one-dimensional coherent scattering process in the “scattering region” for electrons coming from the electrodes. The electronic properties are profoundly susceptible to the confinement region at the nanoscale level. The position and length of the electrodes, as well as the lead regions, are important parameters to obtain the solicited outcome from the device. To understand the reasonable tuning of the spin-polarized electronic conductance between various extremes in the fabrication, we varied the lead length of the gold electrodes. The fluctuation in the electronic transmission was analyzed with the single atom increment in the lead length, and the results are shown in Fig. 7. This highlights the atomic level precision associated with the controlled manipulation of electronic transport through the proposed device. Although our predictions are based on computational simulation, a few successful attempts for the experimental realization of such device fabrication are well documented.^20–22^ The electronic perturbations in the proposed device were induced by increasing the scattering length by adding single Au atoms in the lead region (from three to six) for each step.

The spin-polarized electronic transmission through the molecular junction is susceptible to the magnitude of the scattering region. In our model system, the spin-polarized transmission was observed for the configuration consisting of three and four Au atoms in the lead segment. In Fig. 7, the red and green lines depict the electronic transmission through the alpha and beta spin-channels, respectively, in the high-spin state. Similarly, the blue line corresponds to the transmission in the low-spin state. There is less variation in electronic transmission for the first two systems. However, we saw a considerable increase in the characteristic transmission from the beta electrons in both instances. On the other hand, the spin-polarized electronic transmission disappeared for the lead comprising five and six Au atoms in the device architecture. The transmission behavior was also more or less comparable to that of the low-spin state for a relatively longer scattering region. We can argue that the increase in the scattering length is responsible for the disappearance of the existing spin-polarized electronic transport in the HS state. This particular information could be crucial for device fabrication. Hence, our model configuration of the template for assembling atomic chains accounts for the higher precession in the segment to withstand the spin-polarization effect.

**Fig. 8:**
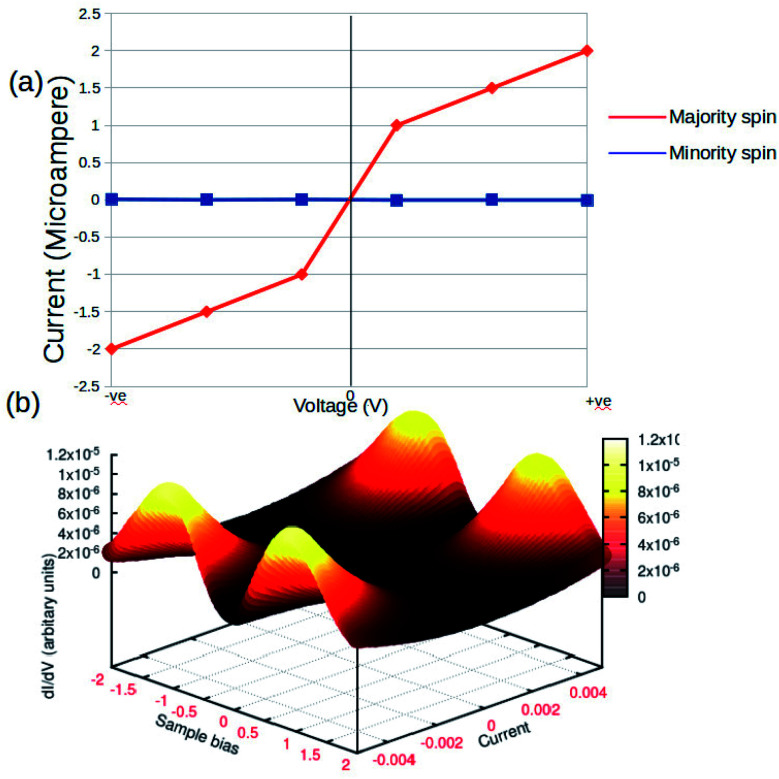
Current–voltage (*I*–*V*) characteristics of Au–Fe-phen–Au in standard (a) 2D graphical format. (b) 3D response surface plots showing the impact of the voltage and current.

The concurrent variations in the conductance and the spin-states established a higher ease of the multifunctional spintronic capability of the Fe-phen system in molecular electronics. To simulate the physical condition in molecular devices working under a finite bias, we computed the current (*I*) through the molecular junction by varying the source-drain bias voltage (VSD). The spin-polarized *I*–*V* trajectory of the Au–Fe-phen–Au molecular junction against the applied voltage is depicted in Fig. 8. The plots clearly show the linear dependence of the current with respect to the applied bias voltage within the particular range. Perhaps the present analytical model envisioned the appreciably large electronic current through one of the spin channels, as compared to that of another spin. In principle, the current associated with one of the electronic spins indicates a nominal fluctuation over the entire spectrum of the bias voltage. This provides exciting implications for the possible spin-filtering capacity of the Fe-phen molecule in the HS state. In the forthcoming study, we will examine the spin filtering performance of Fe-phen and several identical metal complexes with a firm emphasis on the experimental realization of the proposed device.

## Summary and conclusions

In conclusion, we studied the electronic transport behavior through a well-defined molecular junction containing a switchable Fe(ii) spin-crossover complex. The finite bias conductance through the system was monitored by completing the circuit with mechanically controllable atomic gold electrodes. The unfolding of the atomic gold electrodes induced structural deformation in the complex, and triggered the LS-to-HS transition in the system. The present study offered some fresh insights to synchronize the electronic perturbations during the SCO, and devise a futuristic switching device that manipulates the spin-polarized electronic conductance. Furthermore, to the best of our knowledge, this is one of the very first reports describing a systematic analysis on the explicit length-scale of the scattering region to sustain the spin-polarized electronic conductance for the atomic gold electrodes. In the hypothetical nanoscale model, the device could be assembled on some polymer surface, and the subsequent stretching of the surface would introduce the SCO. The effect of the spin transition would modify the electronic transport phenomena and operate as a spin filter. We anticipate that our proposed nanoscale spintronic device architecture will develop genuine interest among the experimentalists, opening a new avenue to design single-molecule organic spin filters in the future.

## Computational methods

We screened five different DFT functionals (PBE,^23^ wB97xD,^24^ B3P86,^25^ B3LYP,^26^ and M06-2X (ref. 27)) to optimize the complex structure without any constraints using the TZP basis set,^28^ as implemented in the ADF 2019.105 software^29,30^ package. There were no significant structural variations observed at the different DFT optimization level for the complex. The optimized geometries and relative energies for the LS and HS states in the B3LYP, CAM-B3LYP, and M06-2X methods are reported in the ESI section.† However, the dispersion corrected (D3) (ref. 31) PBE (Perdew–Burke–Ernzerhof)^32^ PBE(D3) optimized geometry reproduced the energetically favorable reported spin state order for the system. The ADF-BAND utility was used to compute the self-consistent DFT-NEGF-based transport parameters for the molecules attached to an atomic gold chain. Subsequently, the non-equilibrium transport calculations were performed at the same level of theory, *i.e.*, PBE(D3) using the SZ and TZP basis sets, for the gold and constituent atoms in the molecular system, respectively.

## Conflicts of interest

There are no conflicts of interest to declare.

## Supplementary Material

NA-002-D0NA00285B-s001
