# Supplementary material for: Mechanical force-induced manipulation of electronic conductance in a spin-crossover complex: a simple approach to molecular electronics
Source: Nanoscale Adv. 2020 May 14;2(7):2907–13. doi: 10.1039/d0na00285b (PMC9417810; doi:10.1039/d0na00285b)
Supplement: NA-002-D0NA00285B-s001 [file NA-002-D0NA00285B-s001.pdf]

Electronic Supplementary Material (ESI) for Nanoscale Advances.
This journal is © The Royal Society of Chemistry 2020

Supporting Information

Mechanical-Force Induced Manipulation of Electronic Conductance in a Spin-Crossover Complex: A Simple Approach to the Molecular Electronics

Amrit Sarmah,[a,b]* Pavel Hobza[a,b]

[a]Institute of Organic Chemistry and Biochemistry of the Czech Academy of Sciences, Flemingovo nam. 2, CZ-16610 Prague 6, Czech Republic

[b]Department of Physical Chemistry, Palacky University, CZ–77146 Olomouc, Czech Republic

*Corresponding author.

E-mail address: amrit.sarmah@marge.uochb.cas.cz (A. Sarmah)

Mobile No: +420731015016

DFT calculations:

Gas-phase calculations were executed using dispersion corrected PBE i.e., PBE(D3) exchange-correlation functional as enforced in ADF 2019.105 software. For the isolated Fe-phen system geometry optimization was performed at PBE(D3)/TZP level. The same level of theory has been maintained for the DFT-NEGF calculations, except considering a single-ζ basis set for gold atoms to secure uniformity in the computations. Eventually, the density functional theory (DFT) approach coupled with the non-equilibrium Green's function (NEGF) technique was employed to illustrate the charge transport through the electrode-single molecule-electrode junction. Each electrode is described by the one-dimensional chain of atomic gold atoms. The transport calculations are done on the optimized geometries of the isolated molecules (HS or LS configurations), sandwiched by the atomic gold electrodes. The scalar relativistic ZORA (Zero Order Regular Approximation) is further granted for the DFT calculations. Theoretical conductance value G was determined from Landauer formula $G = G0 \times \tau(\varepsilon F)$ considering zero-bias approximation where $\tau(\varepsilon F)$ is the transmission function at the Fermi energy and (εF) Fermi energy of the electrode. In the context of the Landauer Formula, where the conductance is expressed as a sum over scattering channels of transmission coefficients, the transmission coefficients describe the probability that an electron

from, let's say, the left lead is transmitted to the right lead. The value of the transmission coefficients for each channel serves as a probability.

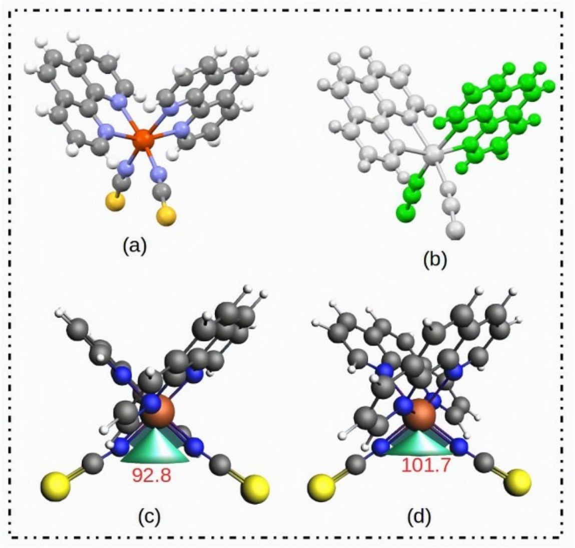

Figure S1: (a) Structure of the Fe(1,10-phenanthroline)$_2$(NCS)$_2$ molecule, (b) 2-fold symmetric orientation represented in different color shades, optimized structure of the complex in low-spin (LS) (c) and high-spin (HS) (d) states at PBE(D3)/TZP level.

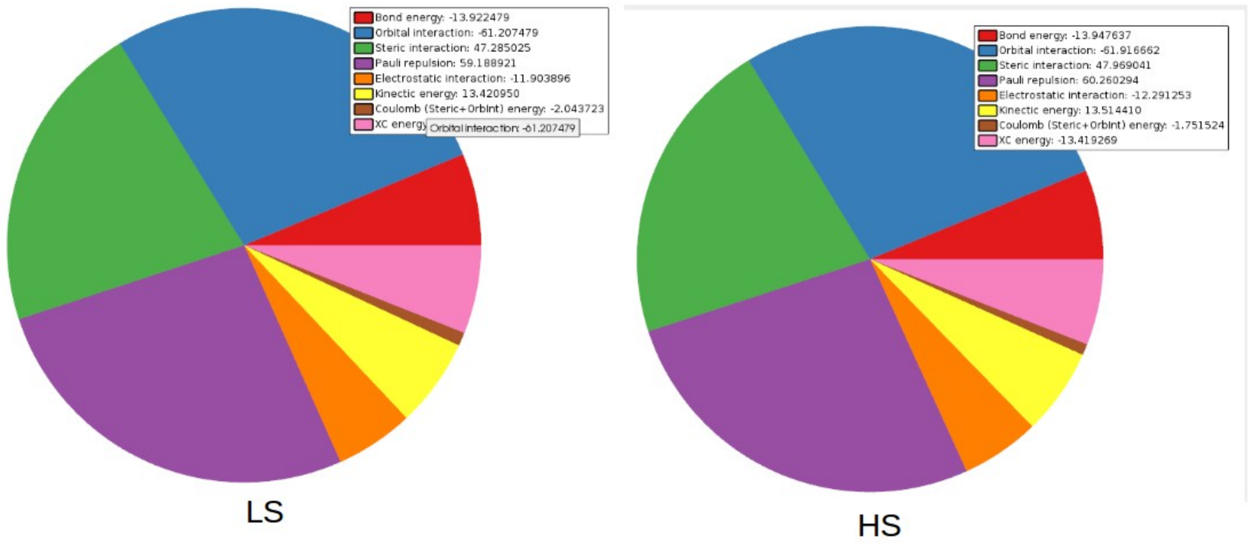

Figure S2: The contribution of the different energy components at PBE(D3)/TZP level obtained from the energy decomposition scheme as implemented in the ADF package for the isolated gas-phase optimized geometry of the complex in the two spin states.

Let's have a look at the relative energetics and relevant structural parameters of the complex in different spin states at different level of theories.

Method	Singlet	Quintet
PBE0	Energy: -3389.10881398<br><br>∠N-Fe-N : 92.80<br><br>S-S bond distance : 7.65Å	Energy: -3389.11454756<br><br>∠N-Fe-N : 101.70<br><br>S-S bond distance : 8.43Å
B3LYP	Energy: -3389.76315482<br><br>∠N-Fe-N : 94.13<br><br>S-S bond distance : 7.63Å	-3389.77348004<br><br>∠N-Fe-N : 106.70<br><br>S-S bond distance : 8.40Å
CAM-B3LYP	Energy: -3389.08143545<br><br>∠N-Fe-N : 95.05<br><br>S-S bond distance : 7.65Å	Energy: -3389.09308834<br><br>∠N-Fe-N : 108.78<br><br>S-S bond distance : 8.43Å

M06-2X	Energy: -3388.41475509<br><br>∠N-Fe-N : 98.06<br><br>S-S bond distance : 7.80Å	Energy: -3389.16418293<br><br>∠N-Fe-N : 109.20<br><br>S-S bond distance : 8.81Å

There are some minimal changes in the energy as well as the structural deviation in the optimized values for the complex at different levels of theory is evident from the above table. This also provides a logical assessment that except the numerical values, the qualitative trend is similar for reported parameters at different level of theories.

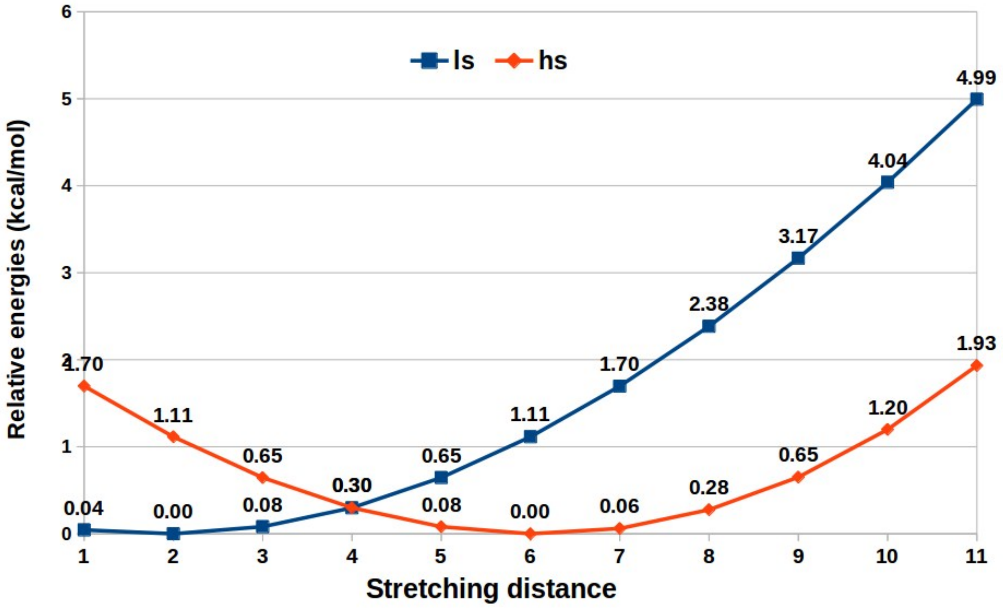

Figure S3: Variation in the structural energy of the LS and HS states as a function of stretching distance.

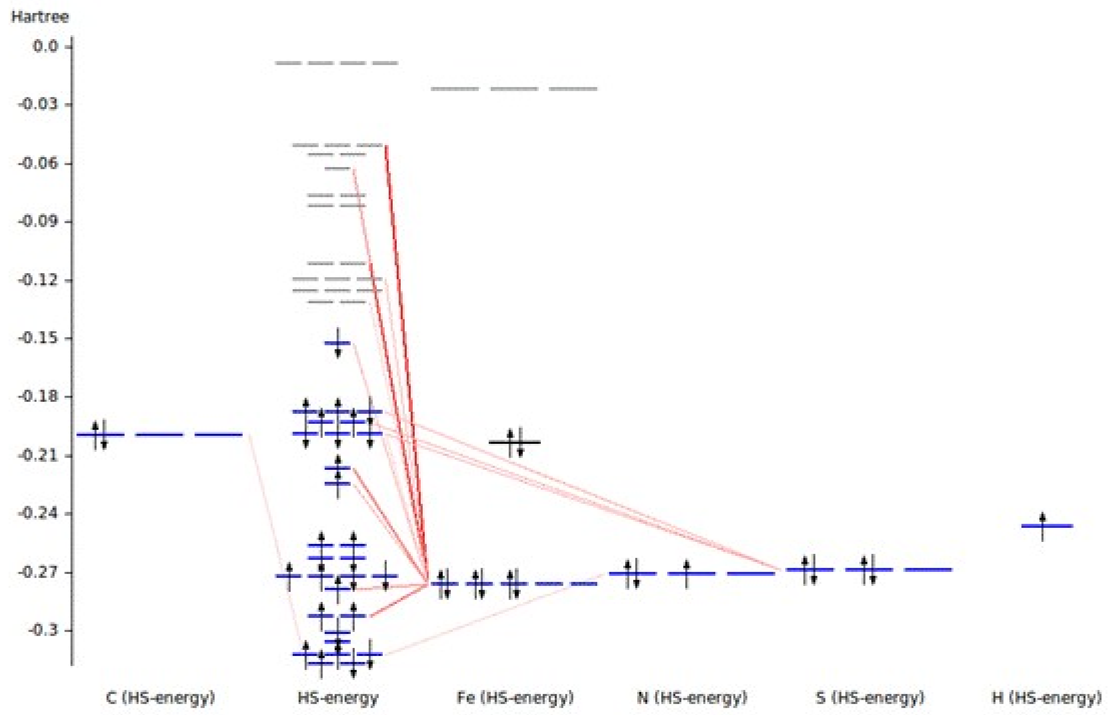

Figure S4: Electronic occupation and the corresponding orbital energy order in the high-spin state of the complex.

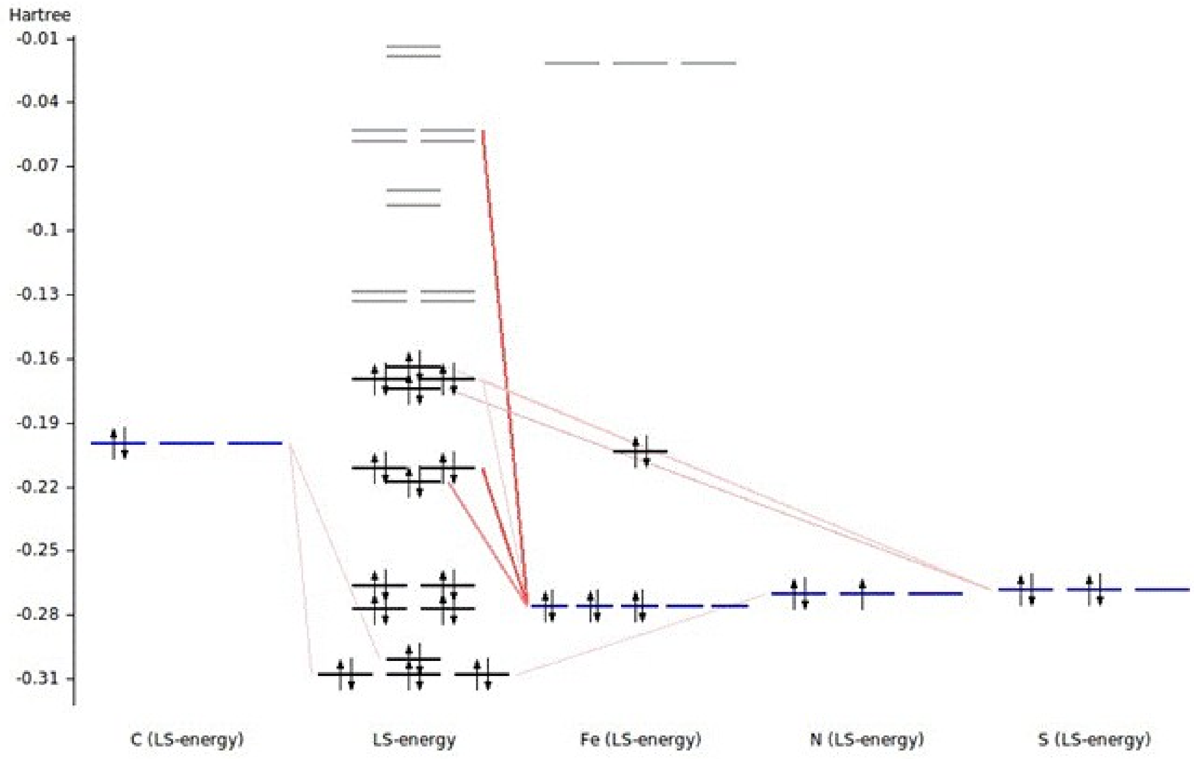

Figure S5: Electronic occupation and the corresponding orbital energy order in the low-spin state of the complex.

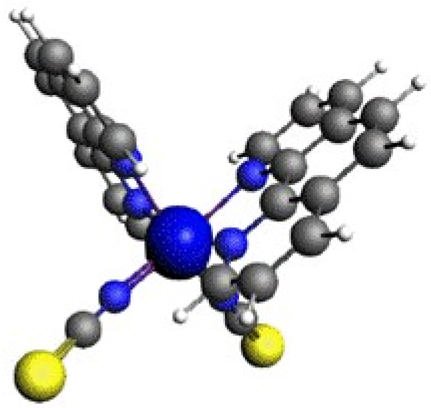

Figure S6: Spin density (η_{UP}-η_{DOWN}) in real space of the isolated SCO Fe^{II} complex in the HS state calculated from DFT.

(a)

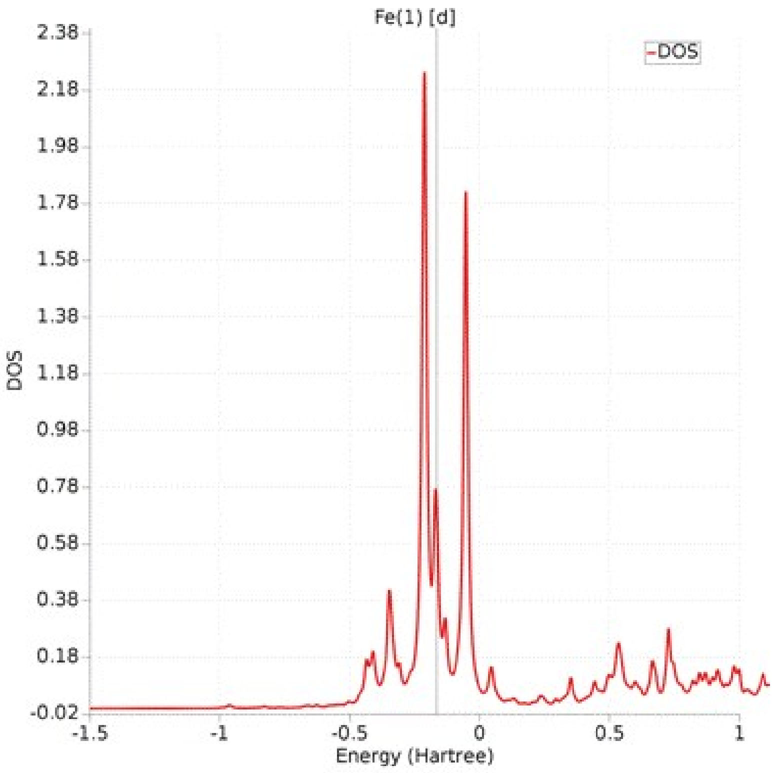

(b)

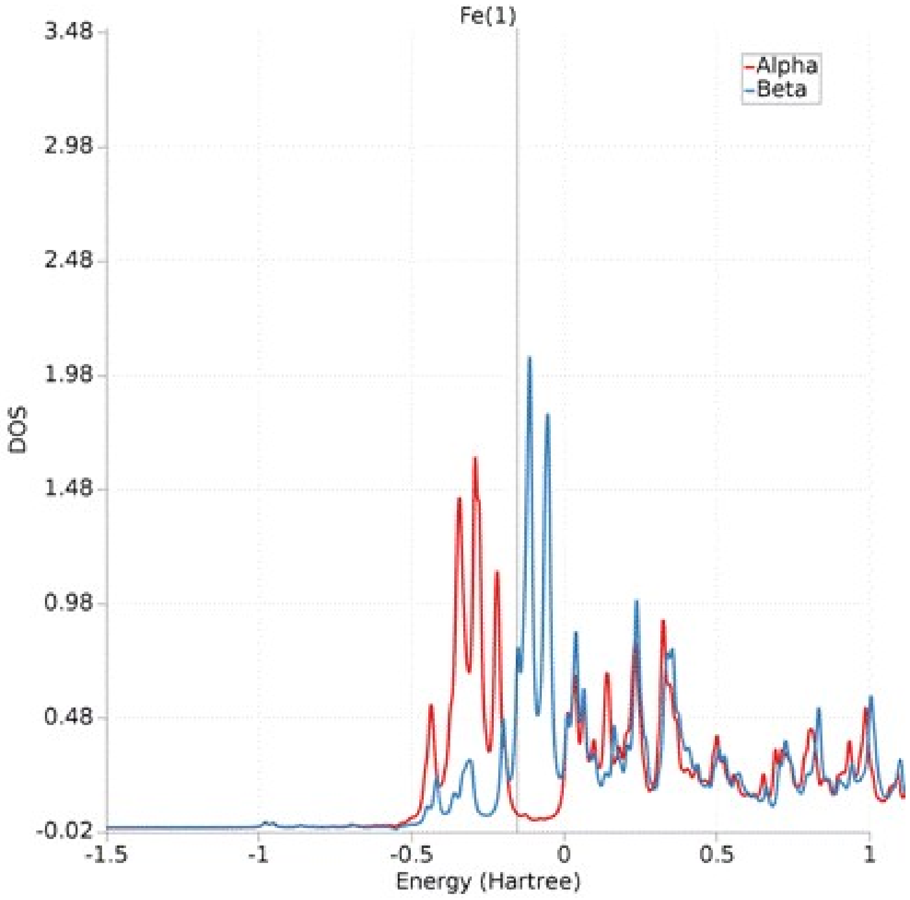

Figure S7: the relative positioning of PDOS for the d-orbitals of Fe atom in (a) low-spin (above) and (b) high-spin (below) states.

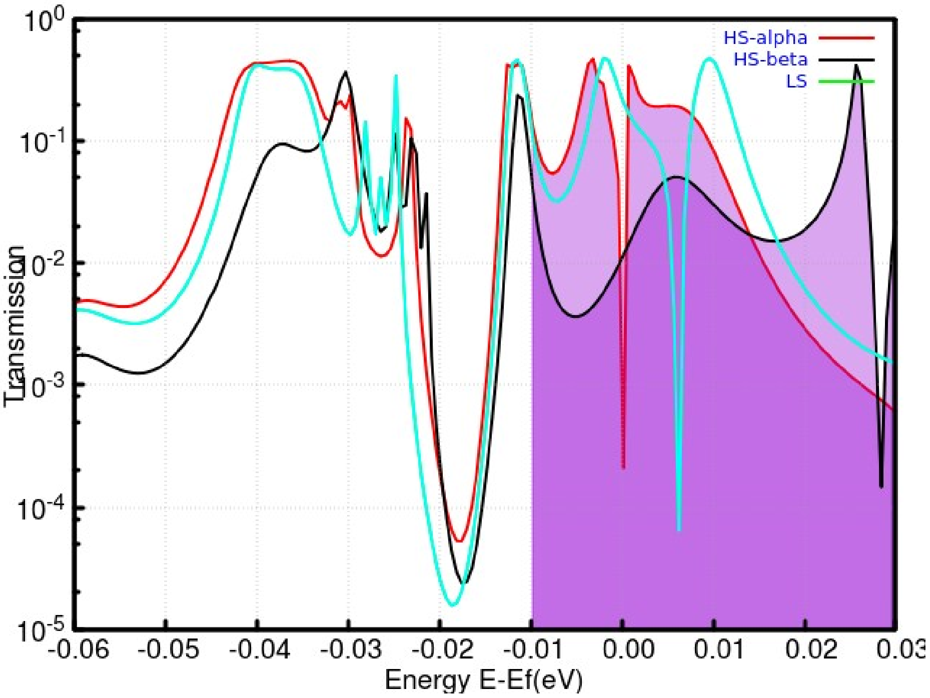

Figure S8: The total transmission of the LS and HS geometries shown in atomic Au electrode based metal−molecule−metal configuration calculated with self-consistent DFT-NEGF. The two different color plots are correspond to spin polarized transmission for up (red) or down (black) spin electrons in the HS state. The electronic transmission in low-spin state is represented by the Cyan colored line in the graph. The Fermi energy of the contacts is at 0 eV.

(a)

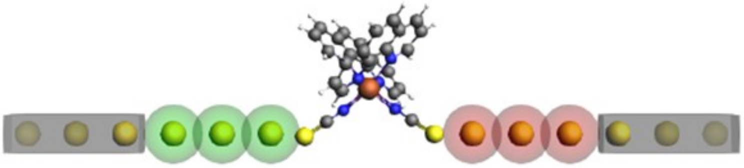

(b)

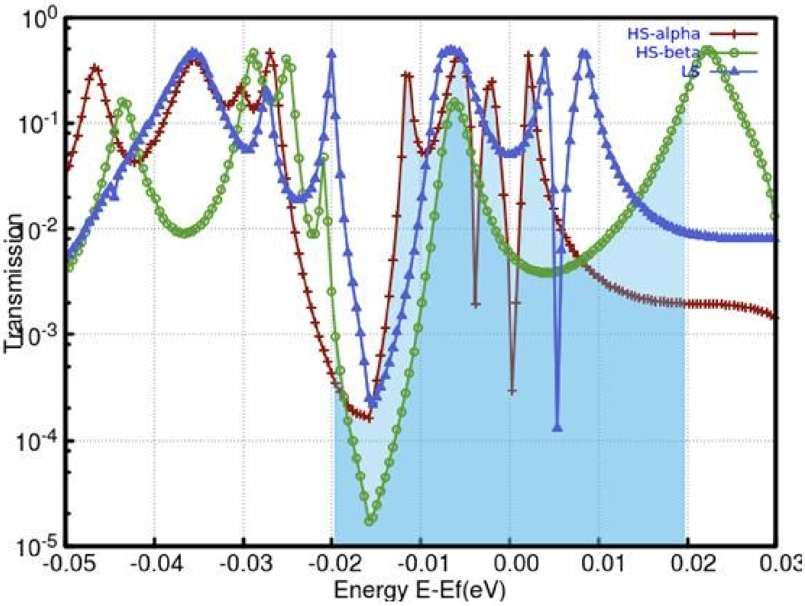

Figure S9: (a) Au electrode based metal−molecule−metal junction considering Au_3 units in the scattering region (b) corresponding total transmission of the LS and HS geometries calculated with self-consistent DFT-NEGF.

(a)

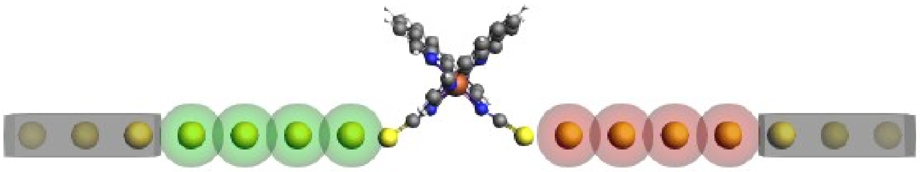

(b)

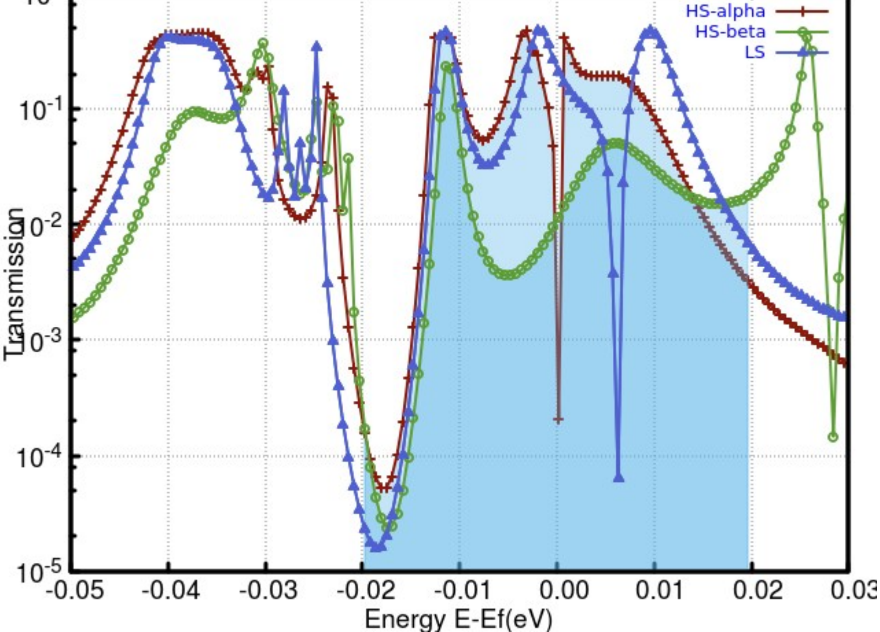

Figure S10: (a) Au electrode based metal−molecule−metal junction considering Au_4 units in the scattering region (b) corresponding total transmission of the LS and HS geometries calculated with self-consistent DFT-NEGF.

(a)

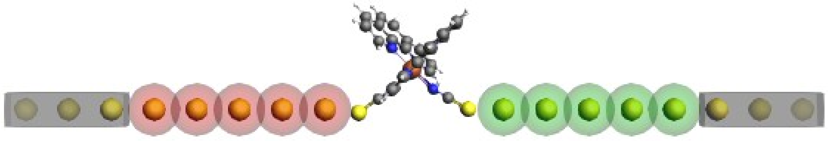

(b)

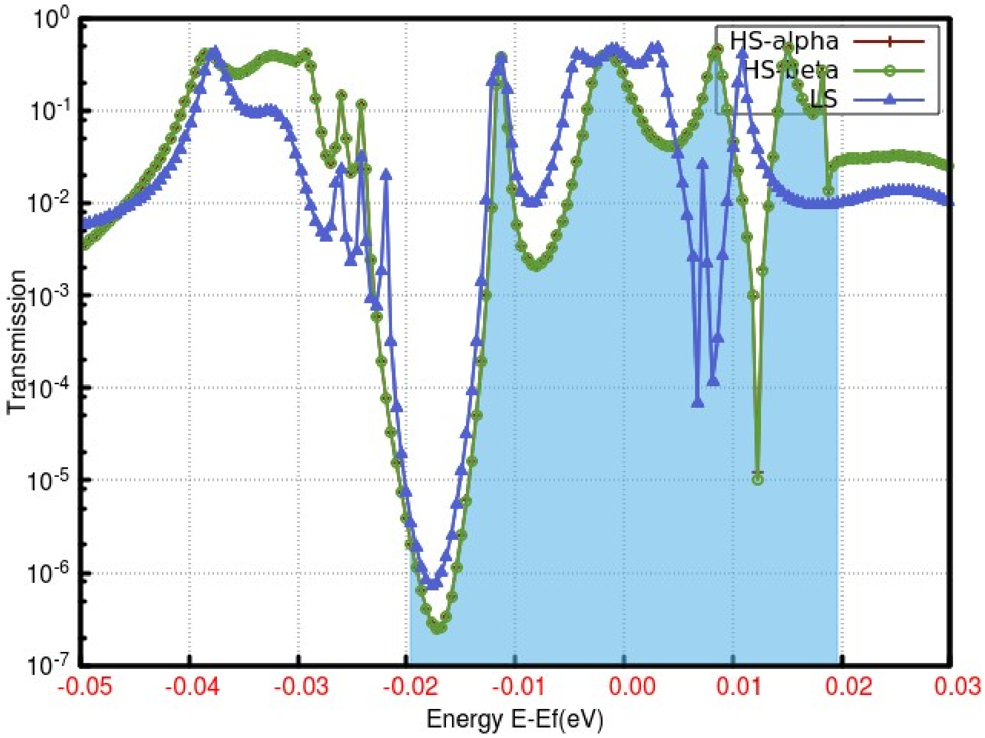

Figure S11: (a) Au electrode based metal−molecule−metal junction considering Au_5 units in the scattering region (b) corresponding total transmission of the LS and HS geometries calculated with self-consistent DFT-NEGF.

(a)

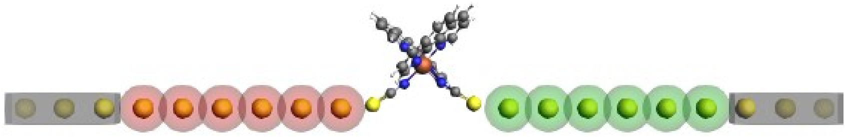

(b)

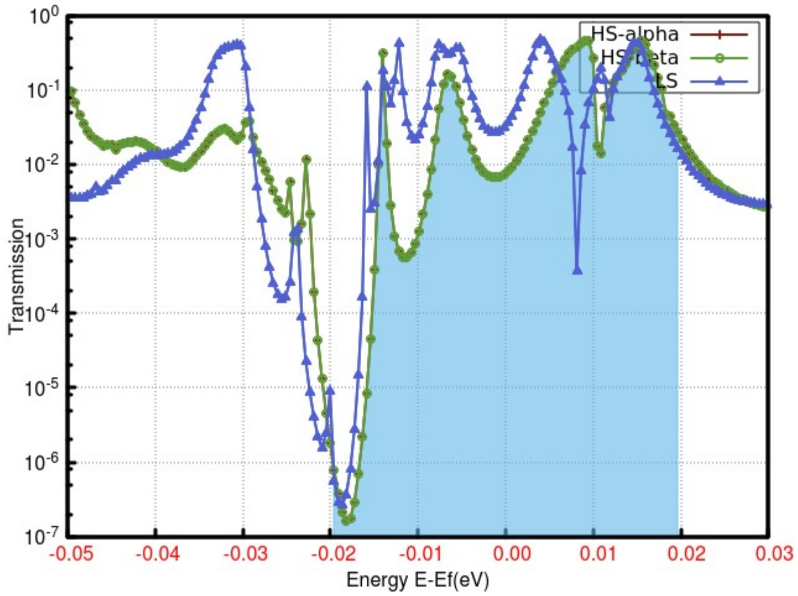

Figure S12: (a) Au electrode based metal−molecule−metal junction considering Au_6 units in the scattering region (b) corresponding total transmission of the LS and HS geometries calculated with self-consistent DFT-NEGF.

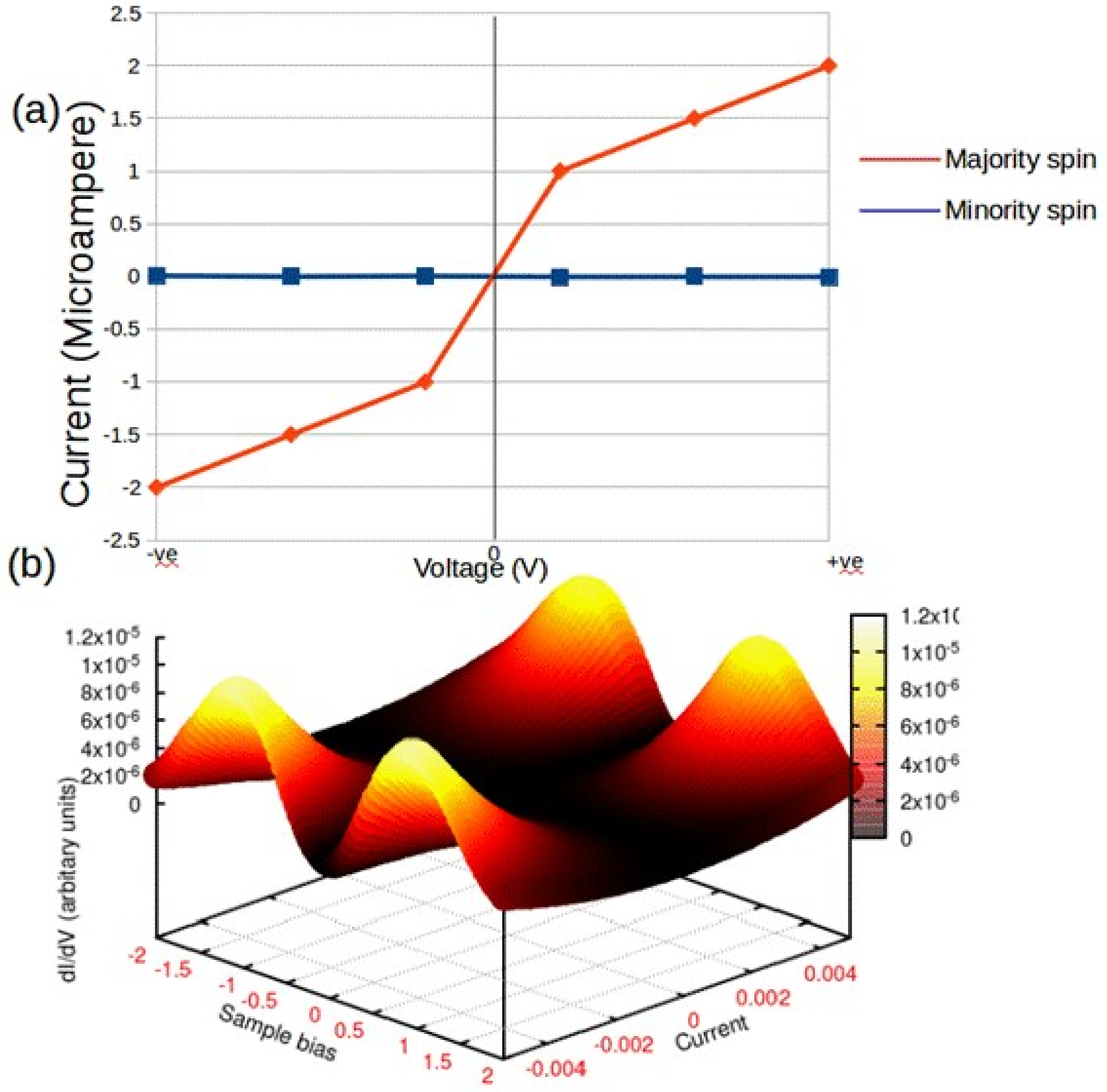

Figure S13: Current-voltage (I-V) characteristics of Au-FePhen-Au in standard (a) 2D graphical format, (b) 3D response surface plots showing the impact of voltage and current.

S14: Gas-phase PBE optimized xyz coordinates of the complex in (a) low (b) high-spin states.

(a)

Fe	6.381041115	1.393222266	4.272137625
N	6.634768465	2.720080097	2.901526726
C	7.147177588	3.358398589	2.044622991
S	7.870839160	4.240269980	0.891036349

N	6.278410612	0.000782075	5.650915746
N	8.303426159	1.327830417	4.687847440
C	7.500796628	-0.329760220	6.161811779
C	8.601898358	0.400512430	5.646000657
C	5.207471041	-0.642207087	6.129968589
C	5.305098686	-1.642212453	7.109405538
C	6.543796732	-1.994675868	7.623511062
C	7.692936679	-1.324211748	7.151304155
C	9.029546400	-1.570608272	7.609850773
C	10.091425303	-0.859072579	7.119622880
C	9.912509723	0.156157587	6.122563026
C	10.951065515	0.941121689	5.576805404
C	10.635732594	1.896384617	4.624041171
C	9.305954392	2.068818982	4.201415184
H	4.246720107	-0.341967874	5.729098501
H	4.396598781	-2.122559199	7.459683018
H	6.637301588	-2.764447129	8.385016486
H	9.183673081	-2.334576803	8.368053796
H	11.097057450	-1.052688090	7.485270056
H	11.975351712	0.796210613	5.910859556
H	11.402804534	2.529217378	4.188645239
H	9.043615620	2.811240749	3.457572689
N	6.127336677	2.719993706	5.642757222
C	5.615790236	3.357166180	6.501064566

S	4.893297253	4.237645057	7.656422612
N	6.483561861	0.000719300	2.893413758
N	4.458660578	1.327862975	3.856603696
C	5.261107521	-0.329907846	2.382753639
C	4.160055950	0.400392513	2.898631804
C	7.554424479	-0.642462427	2.414441367
C	7.456634704	-1.642751625	1.435304230
C	6.217860827	-1.995279188	0.921409649
C	5.068799389	-1.324626155	1.393547224
C	3.732112950	-1.571146581	0.935287233
C	2.670285441	-0.859582505	1.425606272
C	2.849347254	0.155826881	2.422468506
C	1.810835449	0.940682595	2.968480328
C	2.126305487	1.896075486	3.921077141
C	3.456185564	2.068765638	4.343267765
H	8.515219128	-0.342154198	2.815169495
H	8.365061776	-2.123281815	1.085083313
H	6.124240633	-2.765287578	0.160155566
H	3.577882577	-2.335287120	0.177278161
H	1.664581901	-1.053335969	1.060234177
H	0.786464423	0.795563874	2.634783049
H	1.359269437	2.528806247	4.356686561
H	3.718660477	2.811264407	5.086986229

(b)

Fe	6.592655540	1.836532282	4.283670142
N	7.072771383	3.092937263	2.816300797
C	7.691763778	3.736691197	2.028800062
S	8.562939889	4.593186920	0.973798006
N	6.591262728	0.132172617	5.790542266
N	8.721531289	1.369587890	4.703390207
C	7.824194947	-0.324318134	6.135171263
C	8.958571465	0.330503781	5.552677379
C	5.528139153	-0.414580621	6.376262341
C	5.619553040	-1.453068905	7.316457896
C	6.869780577	-1.947533614	7.656959101
C	8.018996934	-1.382360982	7.066971234
C	9.354470530	-1.803035228	7.375569367
C	10.435056996	-1.188384043	6.809970083
C	10.272036123	-0.096267422	5.894958750
C	11.353558449	0.602492088	5.320574942
C	11.097673770	1.668792903	4.471713877
C	9.768248076	2.022195934	4.191614065
H	6.976570249	-2.753835196	8.378789556
H	9.490955804	-2.618158823	8.082700113
H	11.444410520	-1.508408263	7.058679107
H	12.372616701	0.304537382	5.556125335
H	11.904036149	2.235882071	4.017079989

H	9.536540703	2.848034823	3.526421210
H	4.564122424	-0.004881641	6.088268551
H	4.714679559	-1.852611033	7.764246168
N	6.112297852	3.092973874	5.750999889
C	5.493255137	3.736401365	6.538634164
S	4.622038314	4.592838775	7.593702371
N	6.593733069	0.132129320	2.776962292
N	4.463465602	1.369565958	3.864098211
C	5.360799737	-0.324348799	2.432338012
C	4.226424195	0.330487298	3.014834773
C	7.656854003	-0.414610287	2.191222428
C	7.565423975	-1.453084882	1.251034439
C	6.315193674	-1.947547717	0.910564786
C	5.165975272	-1.382383127	1.500551539
C	3.830499776	-1.803054763	1.191966753
C	2.749923025	-1.188395141	1.757579067
C	2.912954842	-0.096277148	2.672580293
C	1.831437585	0.602491404	3.246989007
C	2.087316971	1.668791452	4.095826094
C	3.416778760	2.022178689	4.375874668
H	6.208409004	-2.753816672	0.188716976
H	3.694023434	-2.618155062	0.484803811
H	1.740581530	-1.508408658	1.508832722
H	0.812376934	0.304558867	3.011419296

H 1.280949510 2.235886358 4.550463786

H 3.648491473 2.847999655 5.041076108

H 8.620868150 -0.004932422 2.479230422

H 8.470291401 -1.852619582 0.803236285

S15: Gas-phase B3LYP optimized xyz coordinates of the complex in (a) low (b) high-spin states.

(a)

symmetry c1

C 2.979756000 -3.029305000 1.123130000

C 3.994000000 -2.617149000 0.279182000

C 3.799228000 -1.468621000 -0.518714000

C 2.554873000 -0.812051000 -0.400881000

C 1.769770000 -2.312665000 1.171754000

C 4.773301000 -0.926517000 -1.424519000

C 2.286046000 0.371667000 -1.152253000

C 3.259971000 0.890006000 -2.034810000

C 4.514433000 0.199400000 -2.151071000

C 2.928803000 2.068235000 -2.737097000

H 3.644310000 2.510203000 -3.424024000

C 1.691966000 2.648433000 -2.526297000

C 0.790592000 2.062245000 -1.621831000

H 5.731123000 -1.429869000 -1.516732000

H 3.099731000 -3.898426000 1.760161000

H 4.935272000 -3.156794000 0.230870000

H 0.957780000 -2.596674000 1.830923000

H	5.263741000	0.600011000	-2.827214000
H	1.405910000	3.559188000	-3.040567000
H	-0.175848000	2.514365000	-1.431560000
C	-1.692093000	-2.647884000	-2.526892000
C	-2.928828000	-2.067464000	-2.737703000
C	-3.259913000	-0.889365000	-2.035166000
C	-2.286014000	-0.371369000	-1.152375000
C	-0.790739000	-2.062036000	-1.622197000
C	-4.514274000	-0.198569000	-2.151381000
C	-2.554796000	0.812164000	-0.400688000
C	-3.799073000	1.468897000	-0.518458000
C	-4.773099000	0.927172000	-1.424539000
C	-3.993819000	2.617186000	0.279789000
H	-4.935031000	3.156941000	0.231546000
C	-2.979641000	3.028955000	1.124004000
C	-1.769725000	2.312182000	1.172525000
H	-5.263553000	-0.598900000	-2.827722000
H	-1.406109000	-3.558562000	-3.041340000
H	-3.644314000	-2.509174000	-3.424818000
H	0.175623000	-2.514328000	-1.431938000
H	-5.730852000	1.430658000	-1.516725000
H	-3.099610000	3.897869000	1.761316000
H	-0.957784000	2.595916000	1.831870000
Fe	0.000017000	-0.000187000	0.446788000

N	0.924361000	1.074484000	1.763482000
N	-0.924202000	-1.075138000	1.763317000
C	1.619060000	1.808003000	2.379396000
C	-1.619124000	-1.808622000	2.379010000
S	2.556341000	2.843087000	3.227644000
S	-2.556695000	-2.843682000	3.226962000
N	-1.552899000	1.233922000	0.418890000
N	-1.067031000	-0.947338000	-0.945402000
N	1.552918000	-1.234172000	0.418449000
N	1.066976000	0.947444000	-0.945267000

(b)

symmetry c1

C	3.259489000	-3.058097000	1.077767000
C	4.224773000	-2.616208000	0.194532000
C	3.973114000	-1.471642000	-0.593627000
C	2.718304000	-0.833813000	-0.431365000
C	2.039889000	-2.363145000	1.165798000
C	4.921527000	-0.930038000	-1.525290000
C	2.419618000	0.356659000	-1.187834000
C	3.385942000	0.874601000	-2.087800000
C	4.640170000	0.194101000	-2.241907000
C	3.055180000	2.058685000	-2.781014000
H	3.769263000	2.491126000	-3.476125000
C	1.831167000	2.657091000	-2.556276000

C	0.939706000	2.062747000	-1.645104000
H	5.877573000	-1.432247000	-1.640867000
H	3.423318000	-3.928422000	1.703390000
H	5.176771000	-3.131526000	0.103025000
H	1.254118000	-2.674193000	1.846955000
H	5.368473000	0.601717000	-2.936839000
H	1.550265000	3.573143000	-3.064110000
H	-0.028141000	2.512233000	-1.443891000
C	-1.832300000	-2.653310000	-2.558588000
C	-3.056541000	-2.054895000	-2.782177000
C	-3.387367000	-0.871823000	-2.087309000
C	-2.420899000	-0.354833000	-1.186935000
C	-0.940699000	-2.059961000	-1.646935000
C	-4.641778000	-0.191363000	-2.240107000
C	-2.719587000	0.834487000	-0.428777000
C	-3.974561000	1.472321000	-0.589775000
C	-4.923130000	0.931759000	-1.521896000
C	-4.226220000	2.615749000	0.199991000
H	-5.178337000	3.131018000	0.109487000
C	-3.260688000	3.056586000	1.083526000
C	-2.040968000	2.361767000	1.170251000
H	-5.370218000	-0.598187000	-2.935362000
H	-1.551342000	-3.568641000	-3.067687000
H	-3.770728000	-2.486604000	-3.477637000

H	0.027299000	-2.509502000	-1.446605000
H	-5.879310000	1.433934000	-1.636518000
H	-3.424469000	3.926029000	1.710390000
H	-1.255054000	2.672039000	1.851593000
Fe	-0.000417000	0.000475000	0.749497000
N	0.874196000	1.363416000	1.941496000
N	-0.874134000	-1.364033000	1.940798000
C	1.479888000	2.246833000	2.456139000
C	-1.476911000	-2.250172000	2.454121000
S	2.294604000	3.472791000	3.146454000
S	-2.287564000	-3.479965000	3.142628000
N	-1.772039000	1.286446000	0.432409000
N	-1.216660000	-0.943865000	-0.984215000
N	1.771016000	-1.286845000	0.429475000
N	1.215622000	0.945740000	-0.983946000

S16: Gas-phase CAM-B3LYP optimized xyz coordinates of the complex in (a) low (b) high-spin states.

(a)

symmetry c1

C	-2.954500000	3.039350000	1.109747000
C	-3.968326000	2.624713000	0.279110000
C	-3.780821000	1.474676000	-0.510725000
C	-2.546513000	0.822131000	-0.398075000

C	-1.749690000	2.320163000	1.153828000
C	-4.764890000	0.931848000	-1.404613000
C	-2.286681000	-0.367380000	-1.146950000
C	-3.263842000	-0.883298000	-2.009801000
C	-4.517607000	-0.191730000	-2.121207000
C	-2.945810000	-2.063934000	-2.704847000
H	-3.670699000	-2.505969000	-3.380875000
C	-1.716815000	-2.646491000	-2.505159000
C	-0.807936000	-2.056543000	-1.615615000
H	-5.720432000	1.438904000	-1.490106000
H	-3.067898000	3.912129000	1.741436000
H	-4.908919000	3.164432000	0.232714000
H	-0.932979000	2.606580000	1.805933000
H	-5.271913000	-0.596133000	-2.788109000
H	-1.440028000	-3.561213000	-3.015431000
H	0.159330000	-2.509028000	-1.430646000
C	1.717478000	2.643197000	-2.508532000
C	2.946254000	2.059977000	-2.707651000
C	3.264029000	0.880181000	-2.011063000
C	2.286833000	0.365717000	-1.147382000
C	0.808541000	2.054711000	-1.618084000
C	4.517590000	0.188114000	-2.121661000
C	2.546473000	-0.822763000	-0.396804000
C	3.780627000	-1.475741000	-0.508621000

C	4.764694000	-0.934481000	-1.403464000
C	3.968005000	-2.624588000	0.282974000
H	4.908472000	-3.164585000	0.237270000
C	2.954224000	-3.037696000	1.114427000
C	1.749567000	-2.318190000	1.157564000
H	5.271905000	0.591355000	-2.789256000
H	1.440910000	3.557339000	-3.019960000
H	3.671174000	2.500883000	-3.384381000
H	-0.158560000	2.507734000	-1.433573000
H	5.720096000	-1.441901000	-1.488354000
H	3.067531000	-3.909507000	1.747467000
H	0.932887000	-2.603446000	1.810215000
Fe	0.000015000	0.000491000	0.440447000
N	-0.939412000	-1.071879000	1.746692000
N	0.939058000	1.074644000	1.745473000
C	-1.581571000	-1.839698000	2.361515000
C	1.581243000	1.843126000	2.359397000
S	-2.454084000	-2.933509000	3.203036000
S	2.453811000	2.937864000	3.199646000
N	1.544388000	-1.242975000	0.412232000
N	1.074785000	0.942958000	-0.952005000
N	-1.544406000	1.243808000	0.410168000
N	-1.074443000	-0.944015000	-0.950945000

(b)

symmetry c1

C	-3.330428000	3.128737000	0.850579000
C	-4.294406000	2.583379000	0.036418000
C	-4.023746000	1.383406000	-0.649923000
C	-2.756308000	0.802627000	-0.461675000
C	-2.094208000	2.474383000	0.976186000
C	-4.977156000	0.736982000	-1.507594000
C	-2.433905000	-0.436463000	-1.128817000
C	-3.395126000	-1.050345000	-1.954197000
C	-4.676968000	-0.427691000	-2.131063000
C	-3.041858000	-2.269054000	-2.563942000
H	-3.758258000	-2.776987000	-3.202557000
C	-1.797297000	-2.805093000	-2.332269000
C	-0.909365000	-2.114558000	-1.492874000
H	-5.950601000	1.198887000	-1.639395000
H	-3.506361000	4.045931000	1.400360000
H	-5.263071000	3.060502000	-0.079089000
H	-1.309063000	2.864376000	1.615981000
H	-5.405561000	-0.915992000	-2.770530000
H	-1.496536000	-3.747752000	-2.774332000
H	0.075980000	-2.516961000	-1.278570000
C	1.932655000	2.462016000	-2.724966000
C	3.150103000	1.840558000	-2.870706000

C	3.448061000	0.707197000	-2.089257000
C	2.459864000	0.265293000	-1.189748000
C	1.014095000	1.941970000	-1.798563000
C	4.699977000	0.007694000	-2.164616000
C	2.725337000	-0.878056000	-0.346925000
C	3.965598000	-1.534264000	-0.437098000
C	4.947519000	-1.064542000	-1.373997000
C	4.180624000	-2.630133000	0.423033000
H	5.127241000	-3.161219000	0.386587000
C	3.193764000	-3.006782000	1.302374000
C	1.985535000	-2.289501000	1.318593000
H	5.450045000	0.362776000	-2.864297000
H	1.675457000	3.343019000	-3.301412000
H	3.887896000	2.216841000	-3.573006000
H	0.047741000	2.414755000	-1.649800000
H	5.899215000	-1.583652000	-1.429917000
H	3.328780000	-3.839266000	1.982851000
H	1.179860000	-2.546244000	1.999204000
Fe	0.010978000	0.124071000	0.721883000
N	-0.908828000	-1.192737000	1.993399000
N	0.841590000	1.597525000	1.806572000
C	-1.441816000	-2.116313000	2.494441000
C	1.415806000	2.518804000	2.269431000
S	-2.152517000	-3.417450000	3.169185000

S	2.186398000	3.802100000	2.890251000
N	1.758412000	-1.264068000	0.515610000
N	1.263738000	0.878295000	-1.060081000
N	-1.814120000	1.350862000	0.337320000
N	-1.210562000	-0.967127000	-0.915529000

S16: Gas-phase M06-2x optimized xyz coordinates of the complex in (a) low (b) high-spin states.

(a)

symmetry c1

C	2.974879000	-3.138989000	1.059652000
C	3.982042000	-2.696805000	0.233327000
C	3.798131000	-1.510182000	-0.506963000
C	2.570332000	-0.846172000	-0.353878000
C	1.778592000	-2.401496000	1.151679000
C	4.788861000	-0.954043000	-1.387865000
C	2.320955000	0.376430000	-1.072327000
C	3.312237000	0.905260000	-1.915888000
C	4.557210000	0.201917000	-2.060228000
C	3.021398000	2.119170000	-2.569981000
H	3.762043000	2.566428000	-3.226487000
C	1.805473000	2.725668000	-2.355359000
C	0.877278000	2.114243000	-1.494040000
H	5.734415000	-1.476382000	-1.496535000
H	3.082539000	-4.042297000	1.648170000
H	4.917389000	-3.242626000	0.148068000

H	0.965200000	-2.714040000	1.800352000
H	5.312967000	0.621857000	-2.716718000
H	1.553553000	3.666526000	-2.829552000
H	-0.086184000	2.574332000	-1.295048000
C	-1.805728000	-2.726870000	-2.353632000
C	-3.021694000	-2.120498000	-2.568368000
C	-3.312387000	-0.906182000	-1.914962000
C	-2.320939000	-0.376847000	-1.071918000
C	-0.877337000	-2.114898000	-1.492912000
C	-4.557376000	-0.202917000	-2.059517000
C	-2.570205000	0.846148000	-0.354104000
C	-3.798001000	1.510109000	-0.507431000
C	-4.788883000	0.953474000	-1.387845000
C	-3.981782000	2.697184000	0.232168000
H	-4.917119000	3.242986000	0.146682000
C	-2.974508000	3.139828000	1.058109000
C	-1.778257000	2.402319000	1.150474000
H	-5.313257000	-0.623253000	-2.715612000
H	-1.553921000	-3.668036000	-2.827274000
H	-3.762492000	-2.568160000	-3.224427000
H	0.086179000	-2.574853000	-1.293863000
H	-5.734437000	1.475777000	-1.496687000
H	-3.082059000	4.043500000	1.646087000
H	-0.964803000	2.715204000	1.798908000

Fe	0.000088000	0.000190000	0.547021000
N	0.968044000	1.147543000	1.850190000
N	-0.967922000	-1.146730000	1.850568000
C	1.423076000	2.150435000	2.262525000
C	-1.423169000	-2.149656000	2.262599000
S	2.001426000	3.581497000	2.805083000
S	-2.001828000	-3.580761000	2.804693000
N	-1.581812000	1.291062000	0.458617000
N	-1.119015000	-0.971597000	-0.876537000
N	1.582025000	-1.290668000	0.459174000
N	1.119099000	0.971326000	-0.877001000

(b)symmetry c1

C	3.384685000	-3.011483000	0.987565000
C	4.290979000	-2.459874000	0.122397000
C	3.979614000	-1.257365000	-0.534976000
C	2.727307000	-0.680866000	-0.270719000
C	2.163419000	-2.355903000	1.200885000
C	4.884900000	-0.607396000	-1.434116000
C	2.367231000	0.557662000	-0.916819000
C	3.288481000	1.181773000	-1.770284000
C	4.555982000	0.563651000	-2.020813000
C	2.916012000	2.415307000	-2.335118000
H	3.607075000	2.929792000	-2.990732000

C	1.697193000	2.954734000	-2.029306000
C	0.838178000	2.244792000	-1.176271000
H	5.844640000	-1.071308000	-1.620925000
H	3.589089000	-3.933222000	1.511498000
H	5.248310000	-2.932036000	-0.059868000
H	1.428745000	-2.760046000	1.884847000
H	5.247515000	1.061698000	-2.687896000
H	1.387471000	3.913538000	-2.416977000
H	-0.129097000	2.651477000	-0.908401000
C	-1.690959000	-2.957767000	-2.025417000
C	-2.911009000	-2.421460000	-2.331665000
C	-3.285610000	-1.187472000	-1.769192000
C	-2.365119000	-0.559667000	-0.917626000
C	-0.833001000	-2.244385000	-1.174210000
C	-4.554568000	-0.572494000	-2.020144000
C	-2.727271000	0.679592000	-0.274064000
C	-3.980925000	1.252977000	-0.538725000
C	-4.885513000	0.599127000	-1.435749000
C	-4.294252000	2.456315000	0.116217000
H	-5.252687000	2.926128000	-0.066331000
C	-3.388397000	3.011709000	0.979428000
C	-2.165678000	2.359005000	1.193358000
H	-5.245530000	-1.073507000	-2.685597000
H	-1.379528000	-3.916782000	-2.411183000

H	-3.601423000	-2.938795000	-2.985714000
H	0.135069000	-2.648836000	-0.905886000
H	-5.846359000	1.060648000	-1.622837000
H	-3.594237000	3.934187000	1.501497000
H	-1.431375000	2.766072000	1.876001000
Fe	0.000009000	0.002224000	1.018557000
N	0.767618000	1.524142000	2.251819000
N	-0.771399000	-1.517406000	2.252801000
C	1.001117000	2.656404000	2.055204000
C	-1.004628000	-2.649826000	2.056799000
S	1.256094000	4.227994000	1.719214000
S	-1.259204000	-4.221703000	1.721731000
N	-1.844494000	1.233462000	0.585128000
N	-1.148421000	-1.079635000	-0.652185000
N	1.843996000	-1.231119000	0.590279000
N	1.151678000	1.080553000	-0.651975000
